# Oocyte Casein kinase 1α deletion causes defects in primordial follicle formation and oocyte loss by impairing oocyte meiosis and enhancing autophagy in developing mouse ovary

**DOI:** 10.1038/s41420-022-01184-1

**Published:** 2022-09-17

**Authors:** Di Zhang, Ying Jiang, Xuan Luo, Hui Liu, Yewen Zhou, Sheng Cui

**Affiliations:** 1 College of Veterinary Medicine, Yangzhou University, 225009 Yangzhou, Jiangsu PR China; 2 Jiangsu Co-innovation Center for Prevention and Control of Important Animal Infectious Diseases and Zoonoses, Yangzhou University, 225009 Yangzhou, PR China; 3 Institute of Reproduction and Metabolism, Yangzhou University, 225009 Yangzhou, Jiangsu People’s Republic of China; 4 State Key Laboratory of Agrobiotechnology, College of Biological Sciences, China Agricultural University, 100193 Beijing, People’s Republic of China

**Keywords:** Developmental biology, Physiology

## Abstract

Casein kinase 1α is a member of CK1 family, which is ubiquitously expressed and plays multiple functions, including its potential roles in regulating cell division. But the functions of CK1α in mammalian oogenesis and folliculogenesis remain elusive. In this study, we assayed the cell type of CK1α expression in the developing mouse ovary and confirmed that CK1α was highly expressed in ovaries after birth. The oocyte-specific CK1α knockout (cKO) mouse model was then established by crossing *Ddx4*-Cre mice with *Csnk1a1*-floxp mice, and the effects of CK1α deletion on oogenesis and folliculogenesis were identified. The results showed that oocyte CK1α deletion impaired the progression of oocyte meiosis and primordial follicle formation during meiotic prophase I, which subsequently caused oocyte loss and mouse infertility. Further, the in vivo CK1α deletion and in vitro inhibition of CK1 activity resulted in the defects of DNA double-strand break (DSB) repair, whereas apoptosis and autophagy were enhanced in the developing ovary. These may contribute to oocyte loss and infertility in cKO mice. It is thus concluded that CK1α is essential for mouse oogenesis and folliculogenesis by involving in regulating the processes of oocyte meiosis and DNA DSB repair during meiotic prophase I of mouse oocytes. However, the related signaling pathway and molecular mechanisms need to be elucidated further.

## Introduction

Ovarian follicles are the fundamental functional units in which oocytes are protected and grow. Whereas the formation of primordial follicles is the first stage of folliculogenesis and the foundation for their further development, and the primordial follicle pool established at birth represents the total germ cell population available to a female during her entire reproductive life. In the mouse embryo, germ cells originate from primordial germ cells and migrate to the urogenital ridge [1]. They are referred to oogonia once they colonize the ovary [2, 3]. By 13.5 dpc, oogonia form cysts or nests by mitotic division [4], then enter meiosis initiated by retinoic acid signaling and differentiate into oocytes [5]. The oocytes proceed through prophase I of meiosis, progressing through a series of sub-phases starting with pre-meiotic interphase and eventually arresting at the diplotene stage [3]. Just before or after birth, oocytes undergo a wave of apoptosis, the cysts are broken down by a massive loss of oocytes [6, 7]. While dormant oocytes are surrounded by pre-granulosa cells to form the primordial follicles [3, 8]. The population of primordial follicles is established perinatally, serves as a finite oocyte pool [9], and only a small proportion of primordial follicles are activated concurrently [3, 7].

However, the activation and dormancy of oocytes undergo dynamic alterations in gene expressions, which are regulated by a number of factors, including FOXO3 [10, 11], PTEN [12, 13], PI3K/AKT/mTOR signaling [8], and casein kinase I (CK1) family [14]. In addition, the formation of primordial follicles is dependent on the communication between germ cells and somatic cells established as early as 13.5 dpc [7]. Although there are a number of reports about the molecular events affecting primordial follicle formation and initiating follicle growth, the functions and mechanisms of CK1 family regulating oogenesis and/or folliculogenesis remain elusive.

CK1 is a family of serine/threonine protein kinases [15, 16], which widely exists in eukaryotes, from yeast to humans [17, 18]. Molecular genetic studies have shown that there exist at least seven isoforms of CK1 termed α, β, γ1, γ2, γ3, δ, and ε, and they are greater than 50% identical to one another [15, 16, 19]. The functional studies demonstrate that CK1 kinases are involved in a large number of cellular processes, such as circadian rhythms [20], cellular transformation, mammary carcinogenesis [21], nucleo-cytoplasmic shuttling of transcription factors [22], DNA repair [23], mRNA metabolism [14], cell apoptosis and autophagy [24]. In addition, it is reported that CK1α, encoded by *Csnk1a1* gene, is colocalized with condensed chromosomes during mouse oocyte meiosis and early embryo development [14, 25, 26]. CK1α is thus required for chromosome alignment and segregation during oocyte meiotic maturation by affecting the phosphorylation of Rec8 [27, 28]. These functional studies of CK1α on oocyte meiosis and maturation are mainly performed by using in vitro CK1α knockdown, CK1α RNAi, microinjection of CK1α antibodies, or by using CK1α inhibitors [26, 29]. However, another study shows that CK1, including CK1α, may not be essential for mammalian oocyte meiotic progression [29]. Collectively, although numerous studies have demonstrated the potential roles of CK1 in cell division, the roles of CK1α in the developmental ovary and oocyte maturation are still controversial.

To determine the role of CK1α in oogeneosis and/or folliculogenesis, an oocyte-specific CK1α conditional knockout (cKO) mouse was established in this study. The results firstly demonstrate that oocyte CK1α deletion impairs the meiotic progression of oocytes and formation of primordial follicles, which subsequently cause oocyte loss and mouse infertility by affecting cell apoptosis and autophagy, although the related cellular and molecular mechanisms need to be elucidated further.

## Results

### CK1α expression in the developing mouse ovary

**Fig. 1:**
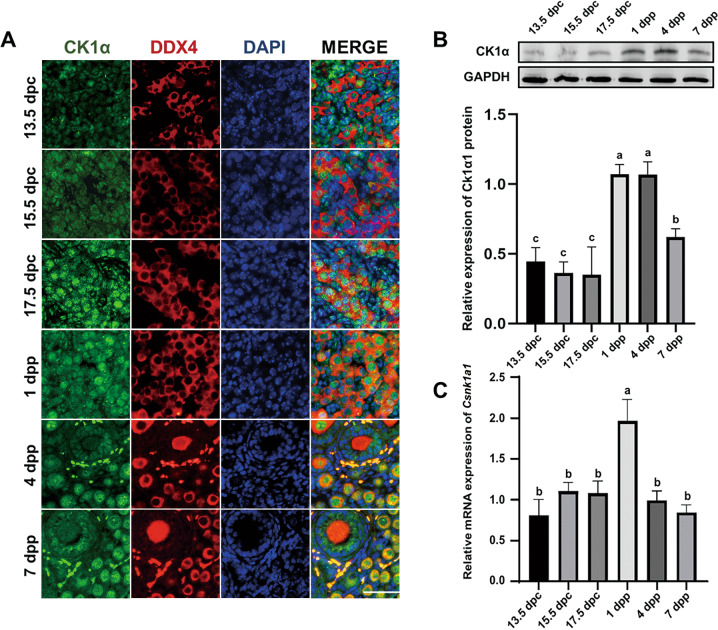
CK1α expression in the developing mouse ovary from 13.5 to 7 dpp. **A** CK1α (green) and DDX4 (oocyte marker, red) dual immunofluorescence in ovaries. Scale bar: 50 μm. **B**, **C** CK1α mRNA and protein levels in different stages of mouse ovaries assayed by RT-qPCR and western blot, respectively. Relative mRNA and protein levels were normalized to GAPDH (*n* = 4). dpc days post-coitum, dpp days post-partum. The same letters indicate the difference is not significant, and the different letters between two bars show a significant difference (*P* < 0.05). The values are the means ± SEM; *P-*values were determined by one-way analysis of variance (ANOVA) with Duncan’s multiple comparisons test.

In order to identify the effects of CK1α on ovary development, we assayed CK1α expression in the developing ovary from 13.5 to 7 dpp. Immunofluorescence (IF) results demonstrated that CK1α was located in the nuclei and widely distributed in the germ cells and somatic cells of newborn mouse ovaries, but CK1α signal was observed both in oocytes cytoplasm and nuclei of 4 dpp and 7 dpp mouse ovaries. The number of CK1α-positive oocytes (labeled by DDX4) and the staining intensity got much more on 1 and 4 dpp (Fig. 1A). Western blot results demonstrated that CK1α protein also maintained at relatively higher levels on 1 and 4 dpp than in other stages examined (Fig. 1B). *Csnk1a1* mRNA level was highly correlated with CK1α protein in the duration examined, with except that *Csnk1a1* mRNA level decreased dramatically on 4 dpp (Fig. 1C). These results demonstrate that CK1α expressions are much higher on 1 and 4 dpp than other stages examined and suggest that CK1α might play important roles in regulating the formation of primordial follicles.

### Establishment of oocyte-specific *Csnk1a1* deletion (cKO) mouse model

**Fig. 2:**
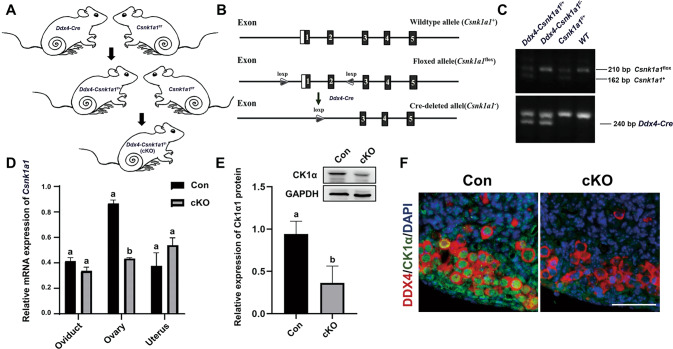
Establishment of oocyte-specific *Csnk1a1* deletion mouse model. **A**, **B** Simplified strategy for creating *Csnk1α1* conditional knockout mouse. **C** Genotype of *Ddx4-Cre* and *Csnk1α1-floxp* assayed by PCR. **D** *Csnk1α1* mRNA levels in the ovaries, oviducts and uteri of 1 dpp mice and **E** Representative image of western blot detecting the knockdown efficiency of CK1α protein and the relative protein levels were normalized to GAPDH (*n* = 3). Different letters represent significant difference (*P* < 0.05). Data are shown as mean ± SEM. **F** Representative images of dual immunofluorescence staining of DDX4 (oocyte marker, red) and CK1α (green) on 1 dpp cKO and control mouse ovaries. DAPI (blue): DNA. Scale bars: 50 μm.

In order to identify the function of CK1α in oocytes, we generated oocytes-conditional CK1α knockout (cKO) mice as the strategy illustrated in Fig. 2A, B, and C [30]. *Csnk1a1* deletion efficiency was assessed in 1 dpp mice ovaries using RT-qPCR (Fig. 2D), western blot (Fig. 2E), and IF staining (Fig. 2F). The results showed that CK1α mRNA and protein levels in the ovary from cKO mice, respectively, decreased by 50 and 40% than the controls (Fig. 2D and E). Whereas *Csnk1a1* mRNA in oviducts and uteri did not exhibit significant differences between cKO mice and controls (Fig. 2D). In addition, CK1α and DDX4 IF dual staining was performed, and it was observed that all DDX4-positive cells were negative for CK1α staining in 1 dpp cKO mouse ovary (Fig. 2F). These demonstrate that CK1α was efficiently deleted in oocytes.

### Oocyte CK1α deletion impairs follicle development and causes mouse infertility

**Fig. 3:**
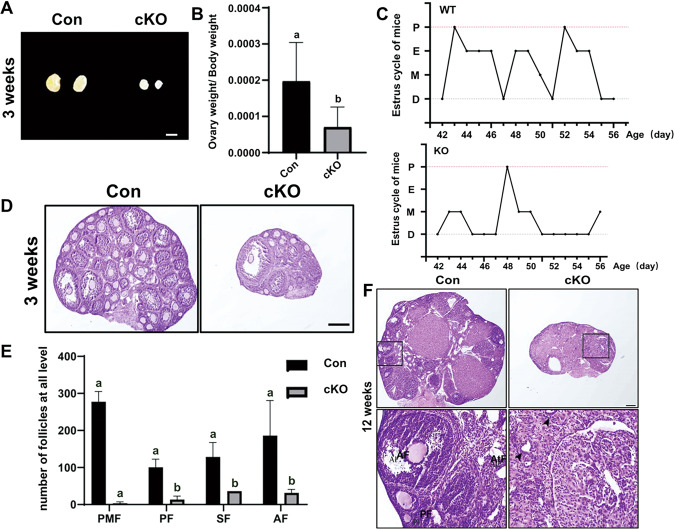
Oocyte CK1α deletion impairs follicle development and causes infertility. **A** Representative images of ovary between control and cKO mice in 3 weeks. Scale bar: 1 mm. **B** The ratio of ovary weight to body weight of mice in 3 weeks. **C** Representative estrous cycle patterns of control and cKO mice in 6 weeks. **D** Histological analysis of ovary from 3 weeks control and cKO mice, Scale bar: 50 μm. **E** Statistical analysis showed the number of different types follicles in 3 weeks ovaries. The different letters (a and b) indicate significant difference (*P* < 0.05). The values are the means ± SEM of three independent experiments. **F** Histological analysis of ovary from 12 weeks control and cKO mice. Scale bars: 200 μm. Arrowheads indicate no oocyte was observed in cKO ovary. PMF primordial follicles, PF primary follicle, SF secondary follicle, AF antral follicle, AtF atretic follicle.

In order to identify the effect of oocyte CK1α deletion on fertility, we conducted an animal breeding assay. The results indicated that the estrous cycles of the cKO mice got disorder (Fig. 3C), and all of them were infertile, although the *Csnk1a1*^f/+^ mice exhibited normal reproductive capacity (Table 1). Further, oocyte CK1α deletion severely impaired ovary growth, and the size of cKO mice was less than 60% of that of control mice (Fig. 3A, B and Supplementary Fig. S1A, B). In addition, morphology and histology examinations were performed on 3 weeks and 12 weeks CK1α cKO and control mouse ovaries. All levels of the follicle, including primary follicle (PF), the antral follicle (AF), and corpus luteum (CL), were clearly observed in control mouse ovaries, but no completed follicle or CL existed in 12 weeks cKO mice (Fig. 3F), although few follicles were detected in 3 weeks cKO mouse ovaries (Fig. 3D and E). These demonstrated that oocyte CK1α deletion impairs follicle development and causes infertility.

**Table 1:** Records of reproduction test of female mice.

Female (*n* = 3)	Male	Pregnancy rate (%)	Average litter size (*n*)
*Csnk1α1*^*f/+*^(Con)	WT	100	7.5 ± 1.24
*Ddx4-Cre; Csnk1α1f/-*(cKO)	WT	0	0

Data were shown as mean ± SEM.

### Oocyte CK1α deletion causes oocyte loss and defect in the formation of primordial follicles

**Fig. 4:**
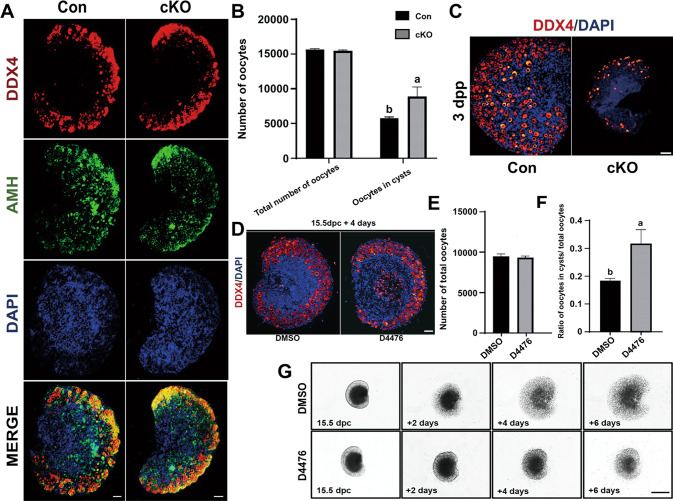
In vivo and in vitro experiments detecting the effects of CK1α inactivation on miotic proliferation, meiosis of the oogonia, and the formation of primordial follicles. **A** Representative images of DDX4 (red) and somatic cell marker AMH (green) immunofluorescent double staining in 1 dpp ovaries. DAPI (blue): nuclei. Scale bar: 50 μm. **B** Number of total oocyte and oocytes in cysts from 1 dpp mice ovaries. Different letters indicate significant difference (*P* < 0.05). Data are shown as means ± SEM (*n* = 3). **C** IF staining of DDX4 (red) in 3 dpp mice ovaries. DAPI (blue): nuclei. Scale bars: 50 μm. **D** DDX4 (red) staining in 15.5 dpc ovaries treated with 25 μM D4476 for 4 days (15.5 dpc + 4 days). DAPI (blue): nuclei. Scale bars: 50 μm. **E** Number of total oocytes and **F** the percentage of oocytes in cysts of 15.5 dpc + 4 days ovaries. Different letters (a and b) indicate significant difference (*P* < 0.05). Values are the means ± SEM (*n* = 3). **G** Embryo ovaries at 15.5 dpc were cultured and treated with D4476 (25 µM) or DMSO (control) for 2, 4, and 6 days, respectively. Scale bar: 100 μm.

As follicle development and oocyte maturation are based on the formation of primordial follicles, the establishment of a primordial follicle pool mainly occurs just before or after birth. We thus analyzed the effects of CK1α deletion on the formation of primordial follicles and afterward follicle development on 1–7 dpp in cKO mice ovary by IF staining. No discernible differences were observed between cKO and control mouse ovaries on 1 dpp (Fig. 4A), but the cKO oocyte number in the cysts was significantly higher than the controls (Fig. 4B). On 3 dpp when the primordial follicle pool has been established, much fewer oocytes and few primary follicles were observed in the cKO ovaries, which accompanied with much smaller ovary size (Fig. 4C). These indicate that oocyte CK1α deletion causes defects in the formation of primordial follicles and oocyte loss.

We further cultured ovaries in vitro, and CK1α activity was blocked with CK1 inhibitor D4476. As the majority of oogonia stop mitosis and entered meiosis from 13.5 dpc, 12.5 dpc ovaries were cultured and treated with 25 μM D4476 or dimethyl sulfoxide (DMSO, control) for 2 days. The results showed that CK1α inhibition did not affect the miotic proliferation and meiosis of the oogonia, which were respectively marked by Ki67 (Supplementary Fig. S2) and synaptonemal complex protein 3 (SYCP3) (Supplementary Fig. S3). Further, 14.5 and 15.5 dpc ovaries were cultured and treated with 25 μM D4476 for 4 days (14.5 dpc + 4 days *or* 15.5 dpc + 4 days). IF staining results showed that D4476 treatment did not have a significant effect on oocytes numbers on 14.5 dpc + 4 days (Supplementary Fig. S4), but on 15.5 dpc + 4 days, the majority of the oocytes failed in the cyst breakdown (Fig. 4D), and the proportion of oocytes in cysts accounting for the total oocytes was significantly higher than the DMSO group (Fig. 4E, F). In addition, 14.5 dpc ovaries were firstly cultured for 3 days for their complete adhesion. DMSO and D4476 were, respectively, added to the medium and cultured for another 6 days, during which the microscope observation and photographing were acquired every 2 days. The results showed that the speed of cell spreading was markedly impaired after D4476 treatment (Fig. 4G). These confirm the in vivo results that CK1α is involved in regulating the formation of primordial follicles and the establishment of primordial follicle pool by affecting the oocyte meiosis and cyst breakdown.

### Oocyte-specific CK1α deletion impairs the meiotic progression and causes abnormalities in DNA double-strand breaks repair

**Fig. 5:**
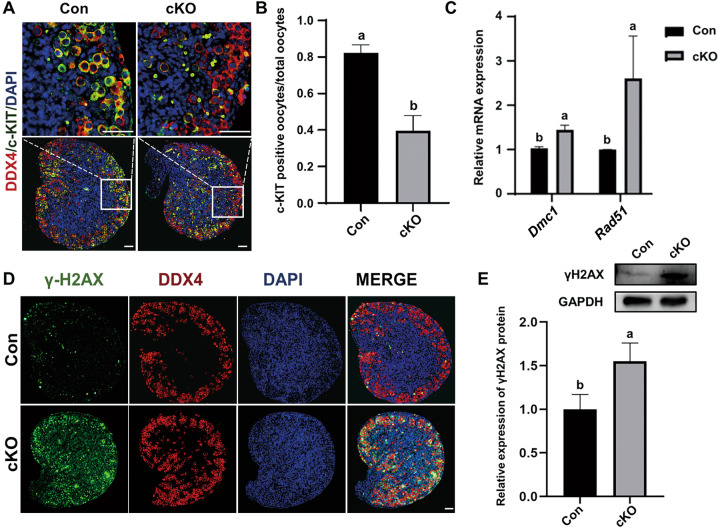
Oocyte-specific CK1α deletion impairs the meiotic progression and causes the abnormalities in DNA double-strand breaks repair. **A** IF staining of DDX4 (red) and c-KIT (green) in 1 dpp mouse ovaries. DAPI (blue): nuclei. Scale bars: 50 μm. **B** Number of c-KIT^+^ oocytes accounting for the total oocytes. Different letters above columns indicate significant difference (*P* < 0.05). **C** RT-qPCR analysis of cell meiosis-related genes. Results were presented as means ± SEM (*n* = 3). Different letters indicate significant difference (*P* < 0.05). **D** γ-H2AX (green) and DDX4 (red) dual staining of 1 dpp mouse ovary. DAPI (blue): nuclei. Scale bars: 50 μm. **E** Western blot analysis of relative γ-H2AX protein levels of mouse ovaries at 1 dpp. Different letters indicate significant difference (*P* < 0.05). The values are the means ± SEM of three independent experiments.

Another characteristic of primordial follicles formation is that most oocytes were impeded at the diplotene stage during meiotic prophase I. Our results showed that the number of c-KIT (a marker of the oocytes in the diplotene stage)-positive oocytes accounting for the total oocytes decreased by 50% in 1 dpp oocytes of cKO mouse ovary (Fig. 5A, B). This implied that CK1α deleted oocytes failed to reach the diplotene stage. In addition, γ-H2AX, an indicator of programmed DNA double-strand breaks (DSBs), and DDX4 dual staining was conducted, and results showed that γ-H2AX signal was much more intensive in 1 dpp cKO ovaries than in the controls (Fig. 5D), which were corresponding to the results assayed by western blot (Fig. 5E). The mRNA expression levels of DSBs repair-associated recombinase *Dmc1* and *Rad51* were assayed by RT-qPCR, and the results showed that *Dmc1* and *Rad51* levels in 1 dpp cKO mouse ovaries were significantly higher than that of controls (Fig. 5C). These indicate that oocyte-specific CK1α deletion impairs the meiotic progression, and causes the abnormalities in DSBs and DSBs repair.

### CK1α deletion enhances the cell apoptosis and autophagy in the mouse ovary

**Fig. 6:**
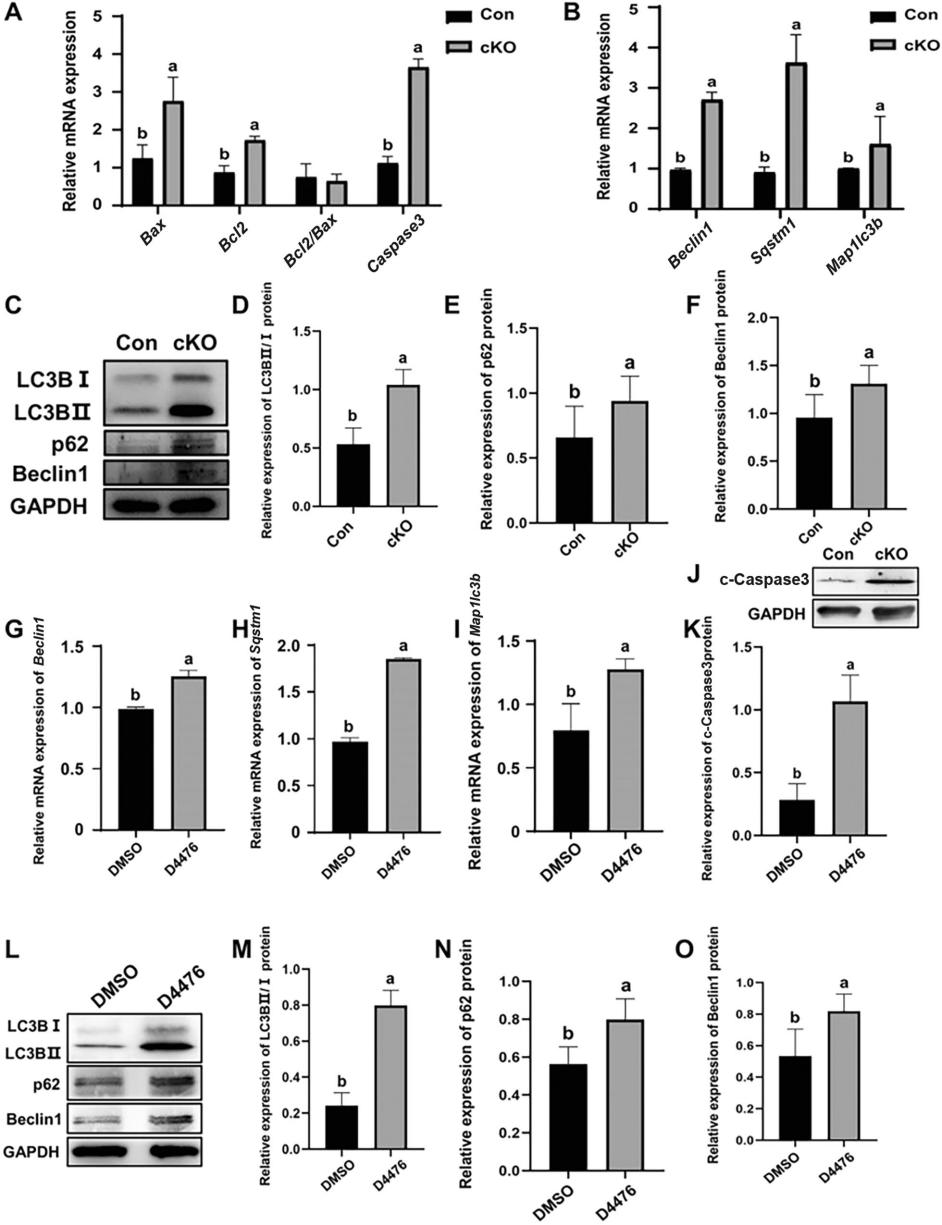
Effects of CK1α inactivation on the gene and protein expressions related to cell apoptosis and autophagy in mouse ovaries. **A**, **B** Respectively, RT-qPCR analysis of apoptotic and autophagy gene expression levels in 1 dpp mouse ovaries. *Map1lc3b* (gene encoding LC3B), *Sqstm1* (gene encoding p62). Results were presented as means ± SEM (*n* = 3). **C** Western blot analysis and **D**–**F** relative expression levels of LC3BII/I, p62, and Beclin1 protein in 1 dpp ovaries. **G**–**I** RT-qPCR analysis of *Map1lc3b*, *Sqstm1*, and *Beclin1* mRNA expression levels in 15.5 dpc embryo ovaries treated with 25 µM D4476 for 4 days (15.5 dpc + 4 days). **J**–**O** Relative expression of cleaved-Caspase3 (c-Caspase3), LC3BII/I, p62, and Beclin1 protein levels in 15.5 dpc + 4 days ovaries detected by western blot. Different letters indicate that the difference is significant (*P* < 0.05). The values are the means ± SEM of three independent experiments.

As oocyte CK1α deletion caused oocyte loss and impaired ovary development, the apoptosis-related gene expression, including *Bcl2*, *Bax,* and *Caspase3* in 1 dpp cKO mouse ovaries was detected. The results showed that oocyte CK1α deletion did not have a significant effect on the ratio of *Bcl2* to *Bax* compared with the controls (Fig. 6A), but sharply increased *Caspase3* mRNA level (Fig. 6A). Further, autophagy-related indicator Beclin1, LC3B and p62 mRNA and protein levels were significantly increased in 1 dpp cKO mouse ovaries compared with the controls (Fig. 6B–F). In addition, the cultured 15.5 dpc mouse ovaries were treated with CK1 inhibitor D4476 for 4 days. The results demonstrated that D4476 significantly increased cell apoptosis (Fig. 6J, K) and the Beclin, LC3B, and p62 mRNA and protein levels than that treatment with DMSO (Fig. 6J, I and L–O) and confirmed the above in vivo results. These collective in vivo and in vitro results demonstrate that the in vivo CK1α deletion and in vitro inhibition of CK1α activity enhances cell apoptosis and autophagy, which may impair the oocyte meiotic process and causes oocyte loss.

## Discussion

In this study, we established an oocyte-specific CK1α deleted mouse model to study the function of CK1α in developing ovaries. The results demonstrate that CK1α is an essential factor for the formation of primordial follicles, follicle development, and oocyte survival by affecting the meiotic progression of oocytes, DSBs repair, and cell apoptosis.

Previous studies have demonstrated CK1 members play multiple functions [17, 31], including their regulating effects on oocyte meiosis [14, 29]. Whereas the mitotic proliferation of oogonia occurs in the early stage of embryo development and is accompanied by entry into meiotic prophase I after 13.5 dpc, followed by cyst breakdown and formation of primordial follicles [32]. The present study demonstrates that CK1α has low expression in the prenatal ovary, but its expression level is much higher in 1 dpp mouse ovary and expresses in both somatic and germ cells when the mitotic proliferation of the oogonia terminates, and oocytes get into the meiotic prophase for follicle formation [3, 8]. Whereas oocyte CK1α deletion impairs oocyte meiosis implied by the decrease of c-KIT positive oocyte number but does not have a significant effect on the mitotic proliferation of oogonia. These imply that CK1α is essential for the progression of oocyte meiosis during meiotic prophase I and the formation of primordial follicles.

Further, the results presented here demonstrate that oocyte CK1α deletion impairs follicle development and causes infertility. Firstly, this may result from the defects in the cyst breakdown and formation of primordial follicles caused by the dysregulated meiosis in the cKO mice as presented in this study. In addition, oocyte CK1α deletion enhances the apoptosis and autophagy indicated by the elevation of the Beclin, LC3BII/I, and p62 expression [33–35], which are confirmed by inhibiting CK1 activity through in vitro experiments also. Just because of the defects in oocyte meiosis, the enhancement of cell apoptosis, and autophagy caused by CK1α deletion, most oocyte get loss, and folliculogenesis are impaired. In support, no completed follicle or corpus luteum exists in 12 weeks cKO mice ovaries, although few follicles are detected in 3 weeks in cKO ovaries. These infer that CK1α is an intrinsic factor for mouse oogenesis and oocyte survival, although the molecular mechanisms under which need to be elucidated.

In addition, there are reports that the programmed DNA DSBs occur during meiotic recombination in the germline cells of mammals [36–39]. During mouse oogenesis, the DNA DSBs occur mainly at the pachytene stage oocytes, and the DNA DSBs are repaired by homologous recombination [40, 41]. The results of the present study show that oocyte CK1α deletion significantly increases the proportion of γH2AX positive oocytes and γH2AX expression, a DNA damage marker. In addition, it has been identified that CK1 kinases, probably involving also CK1α, are essential for initiating apoptosis in response to DNA damage [42, 43]. So, the elevation of γH2AX expression might be due to delayed apoptosis when CK1α is not present. However, DNA repair-associated recombinase RAD51 and DMC1 have similar expressing patterns as γH2AX, and infer that the oocytes may still be capable of initiating a DNA repair response by homologous recombination [44, 45], although oocytes CK1α deletion increases the expressions of Beclin, LC3BII/I, and p62, the molecular markers of autophagy. However, the results of the present study are limited to clarify the effects of oocyte CK1α deletion on the DSBs, cell apoptosis, and autophagy. The relative cellular and molecular mechanism under which need to be elucidated further.

In conclusion, to determine the role of CK1α in oogenesis and/or folliculogenesis, an oocyte CK1α conditional knockout (cKO) mouse was established in this study. The results demonstrate that oocyte CK1α deletion impairs the meiotic progression of oocytes and the formation of primordial follicles, oocyte loss, and infertility by affecting cell apoptosis and autophagy, although the related signaling pathway and detailed mechanism need to be elucidated in further study.

## Materials and methods

### Animals and treatments

Institutional Animal Care and Use Committee (IACUC) at the Yangzhou University approved the experimental protocol of this study. All mice (in C57BL/6 strain) were housed in polyethylene cages under controlled laboratory conditions and provided with standard mice chow and water ad libitum. *Csnk1a1*-floxp/floxp (*Csnk1a1*^*f/f*^) (Stock #025398) [30] mice and *Ddx4*-Cre mice (Stock #006954) [46] were obtained from the Jackson Laboratory (Bar Harbor, ME, USA) and were crossed to obtain *Ddx4*^Cre^*;Csnk1a1*^floxp/−^ (cKO) mice (Fig. 2A). The female cKO mice of 6–8 weeks old were mated with wild-type males at a ratio of 1:1 overnight and checked for the vaginal plug the next day morning. The morning plug was considered as 1 dpc.

### Estrous cycle assessment

Female mice 6 weeks old were used to assess the estrous cycle stage every day using vaginal lavage procedures. Vaginal cytology was analyzed after being stained with Wright’s dye (Solarbio Life Sciences, Beijing, PR China). Mice were determined to be in proestrus when nucleated cells were the predominant cell type, estrus when cornified cells were predominant, metestrus when cornified cells and leukocytes were predominant, and diestrus when leukocytes were predominant [47].

### Breeding assay

Eight weeks cKO and control female mice were bred with wild-type (WT) males for up to 3 weeks. Vaginal plugs were detected every morning, and the females were separative caged when plugged for 18 days or until parturition. The litter size was recorded for each genotype.

### Realtime quantitative PCR (RT-qPCR)

Total RNA was extracted from samples by RNAiso Plus (Takara, Dalian, PR China), and 2 μg of total RNA from samples were reverse transcribed using M-MLV reverse transcriptase (Promega, Madison, WI, USA). RT-qPCR was conducted using SYBR Green master mix (Vazyme Biotech Co. Ltd, Nanjing, PR China) in an ABI PRISM 7500 Sequence Detection System (Applied Biosystems; Thermo Fisher Scientific Corp., Waltham, MA, USA) according to the manufacturer’s protocol using the primers shown in Supplementary Table S1. The mixture was heated to 95 °C for 10 min, followed by 40 cycles of 95 °C for 15 sec and 60 °C for 1 min. Analyses were conducted in triples, and 2^−ΔΔCt^ method was used to determine gene expression levels. Target genes were normalized to GAPDH as endogenous control.

### Western blot

Ovary Proteins were extracted with RIPA buffer (50 mM Tris-HCl, pH 7.4, 150 mM NaCl, 1% Triton X-100, 1% sodium deoxycholate and 0.1% sodium dodecyl sulfate (SDS)) containing 1 mM phenylmethylsulphonyl fluoride (PMSF). After six times of alternant freezing and melting, the samples were incubated on ice for 30 min. Samples were centrifuged at 15,000 × *g* for 30 min at 4 °C. The supernatant containing proteins was transferred to new centrifuge tubes. Protein concentrations were evaluated using a BCA Protein Assay kit (CWBIO, Beijing, China). The protein extracts (~50 μg) were separated by 12% SDS–polyacrylamide gel electrophoresis (PAGE) and electro-transferred to polyvinylidene difluoride (PVDF) membranes (Bio-Rad Laboratories). After blocking membranes in 5% (w/v) non-fat dry milk prepared in 0.05 M Tris-buffered saline Tween-20 (TBST; pH 7.4) for 3 h at room temperature, membranes were incubated with the primary antibodies listed in Supplementary Table S2 overnight at 4 °C. After briefly washing three times, those membranes were incubated with specific secondary antibody (1:20,000) (Zhongshan Bio Corp., Beijing, PR China) at room temperature for 3 h. The relative intensity of each blot was assessed using ImageCal software (Tanon, Shanghai, China). The intensity values corresponding to each sample were analyzed by Image J (National Institutes of Health) and normalized against the density of GAPDH.

### Immunofluorescence staining

Immunofluorescence staining was performed as described previously [48]. Ovaries were fixed in 4% paraformaldehyde overnight at 4 °C, after which the ovaries were embedded in paraffin and sectioned (5 μm). Primary antibodies listed in Supplementary Table S2 were applied to the tissues overnight at 4 °C. Then samples were incubated for 3 h at room temperature with specific secondary antibodies. DAPI was applied to identify the cell nucleus. The signals were collected by using a fluorescence microscope photograph system (Olympus, Tokyo, Japan).

### Ovary isolation and culture

The ovary isolation and culture were performed as reported by Cai et al. [49]. Briefly, the ovaries from 12.5, 13.5, 14.5, or 15.5 dpc mice were separated and washed by prechilled PBS three times, which were then cultured in 6-well culture dishes containing 1 ml of basic DMEM/F12 medium (Gibco, Life Technologies) at 37 °C in 5% CO_2_/95% air atmosphere with saturated humidity. Half of the medium was replaced every 2 days until the ovaries grew to the required stages. 25 μM D4476 was added to detect the effects of CK1α on folliculogenesis.

### Statistical analysis

Data are expressed as the mean ± SEM. Statistical analysis was performed using SPSS 10.0 (SPSS, Inc.). Independent-samples *t*-tests were used to compare the significance of differences between two groups. One-way analysis of variance (ANOVA) was used to compare the significance of differences among multiple groups. Differences were considered significant at two-sided *P* < 0.05. Each experiment was repeated at least three times.

## Supplementary information

Supplementary material

Supplementary Figure 1

Supplementary Figure 2

Supplementary Figure 3

Supplementary Figure 4

Original Data File

## Data Availability

All data generated or analyzed during this stugy are available from the corresponding author on reasonable request.
