# Supplementary material for: Oocyte Casein kinase 1α deletion causes defects in primordial follicle formation and oocyte loss by impairing oocyte meiosis and enhancing autophagy in developing mouse ovary
Source: Cell Death Discov. 2022 Sep 17;8:388. doi: 10.1038/s41420-022-01184-1 (PMC9482644; doi:10.1038/s41420-022-01184-1)
Supplement: Supplementary file 1 — Supplementary material [file 41420_2022_1184_MOESM1_ESM.docx]

**Supplementary Material**

***Figure S1.*** **Oocyte CK1α deletion impairs ovary growth.** A, Representative bright field images of ovary between control and cKO mice in 12 weeks. B, The ratio of ovary weight to body weight of the control and cKO mice in 12 weeks.

***Figure S2.*** **The effect of CK1α inactivation on oogonia miotic proliferation.** Immunofluorescence double staining of Ki67 (green) and DDX4 (red) were conducted in 12.5dpc ovary treated with D4476 for 2 days. DAPI (blue): DNA. Scale bar: 50μm.

***Figure S3.*** **The effect of CK1α inactivation on oogonia meiosis.** Immunofluorescence double staining of SYCP3 (green) and DDX4 (red) were conducted in 12.5dpc ovary treated with D4476 for 2 days. DAPI (blue): DNA. Scale bar: 25μm.

***Figure S4.*** **The effect of CK1α inactivation on oocytes number.** A, Immunofluorescence staining of DDX4 (red) in 14.5dpc ovary treated with D4476 for 4 days (14.5dpc + 4 days). DAPI (blue): DNA. Scale bar: 50μm. B, The number of total oocytes in 14.5dpc + 4 days ovary.

***Table S1* Primer list**

| Genes | Primer sequence (5’-3’) |
| --- | --- |
| *csnk1a1* | F- CTGGCTCTTTCGGGGACATT  R- TGCTCTCGTACAGCAACTGG |
| *dmc1* | F-TTCTGGGTCGGCGTTAGAAT  R-CGGCAGTAAAATAATCAATTCCCGA |
| *rad51* | F-CCAGCTCCTTTACCAAGCGT  R-CACTGCGACACCAAACTCAT |
| *bax* | F-AAACTGGTGCTCAAGGCCC  R-CTTGGATCCAGACAAGCAGC |
| *bcl2* | F-GAACTGGGGGAGGATTGTGG  R-GCATGCTGGGGCCATATAGT |
| *caspase3* | F-GAGCTTGGAACGGTACGCTA  R-GAGTCCACTGACTTGCTCCC |
| *beclin1* | F-TCTCGTCAAGGCGTCACTTC  R-CCCGGTCTTCAGCTACTTCC |
| *p62* | F-AGGAGACCATTGCCAAGTCTTT  R-TGTGACAGGAGCAAGAGTGG |
| *lc3b* | F-GGGACCCTAACCCCATAGGA  R-GGCACCAGGAACTTGGTCTT |
| *gapdh* | F- GCTCACTGGCATGGCCTTCCGTG  R- TGGAAGAGTGGGAGTTGCTGTTGA |

*Table S2* Antibody list

| Antibody | Host | Company (Catalog) |
| --- | --- | --- |
| CK1α | Rabbit | Abcam (ab64939) |
| DDX4 | Mouse | Abcam (ab27591) |
| Ki67 | Rabbit | CST (9129) |
| c-Caspase3 | Rabbit | CST (9661) |
| c-KIT | Rabbit | CST (3074) |
| γ-H2AX | Rabbit | Novus (NB100-384) |
| p62 | Rabbit | Wanleibio (WL02385) |
| Beclin1 | Rabbit | Wanleibio (WL02508) |
| (active) β-catenin | Rabbit | CST (8814) |
| β-catenin | Rabbit | CST (8480) |
| LC3B | Rabbit | CST (3868) |
| SYCP3 | Rabbit | Abcam (ab15093) |
| GAPDH | Mouse | Abclonal (AC002) |
