# Supplementary figures and images for: Oocyte Casein kinase 1α deletion causes defects in primordial follicle formation and oocyte loss by impairing oocyte meiosis and enhancing autophagy in developing mouse ovary
Source: Cell Death Discov. 2022 Sep 17;8:388. doi: 10.1038/s41420-022-01184-1 (PMC9482644; doi:10.1038/s41420-022-01184-1)

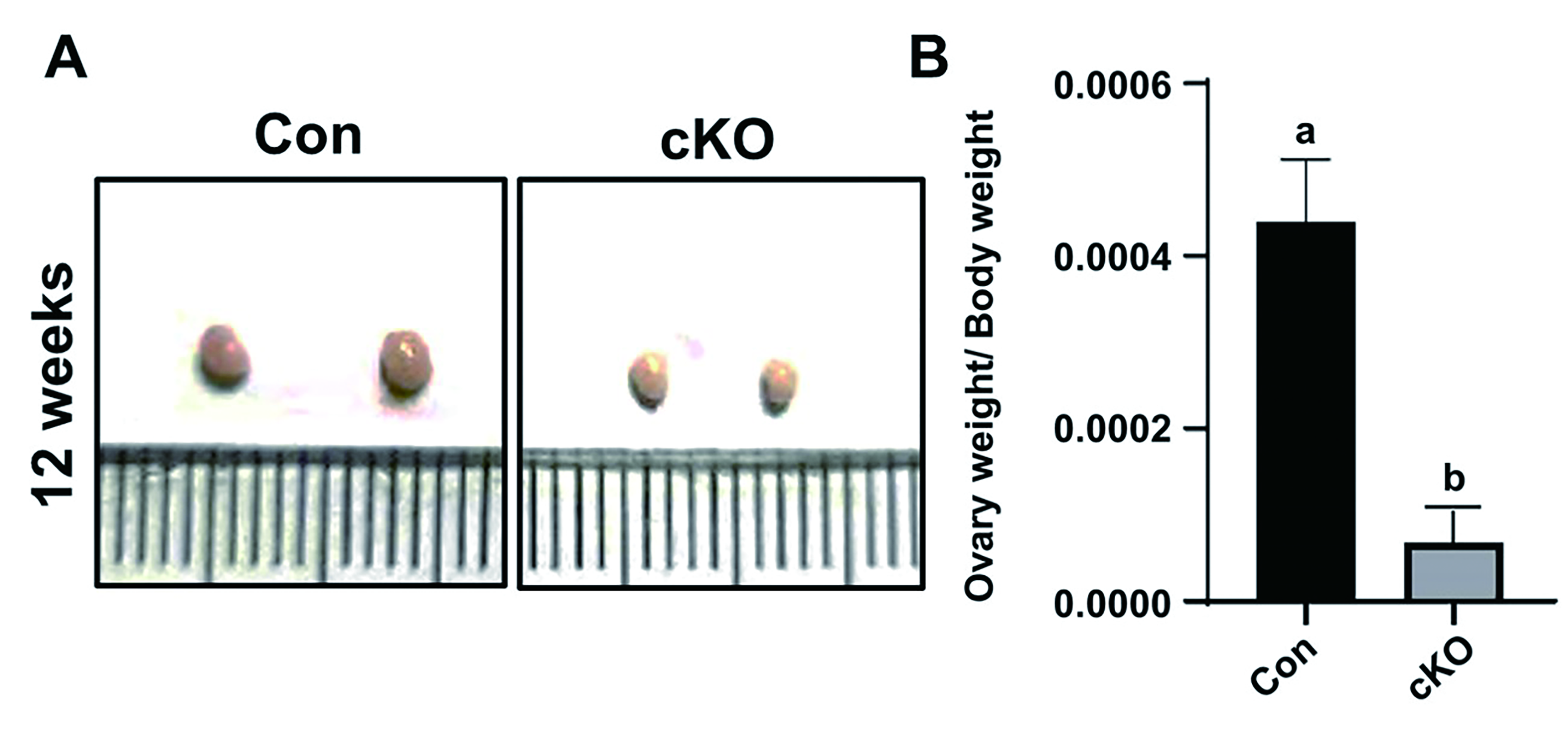

Supplement: Supplementary file 2 — Supplementary Figure 1 [file 41420_2022_1184_MOESM2_ESM.tif]

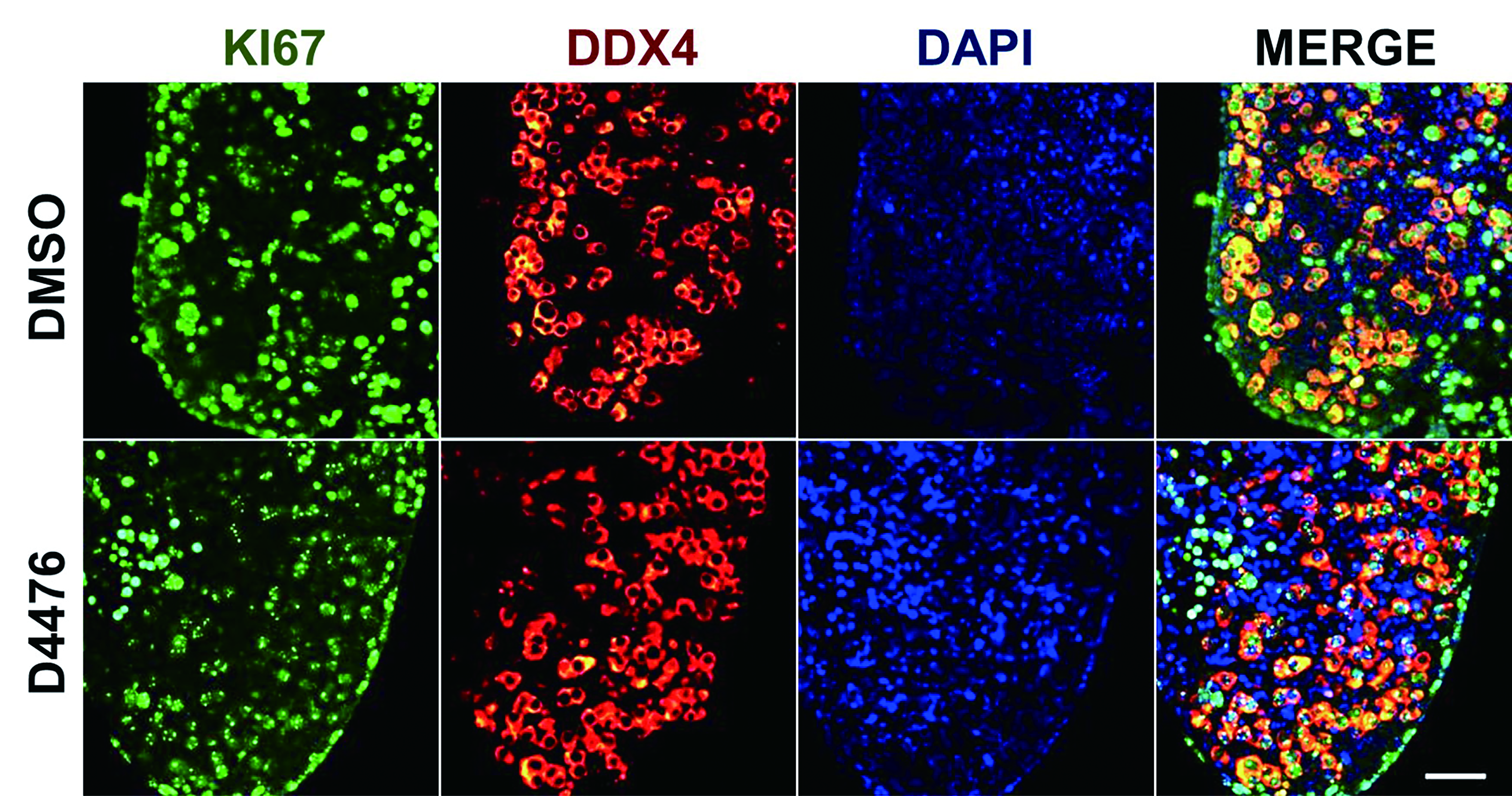

Supplement: Supplementary file 3 — Supplementary Figure 2 [file 41420_2022_1184_MOESM3_ESM.tif]

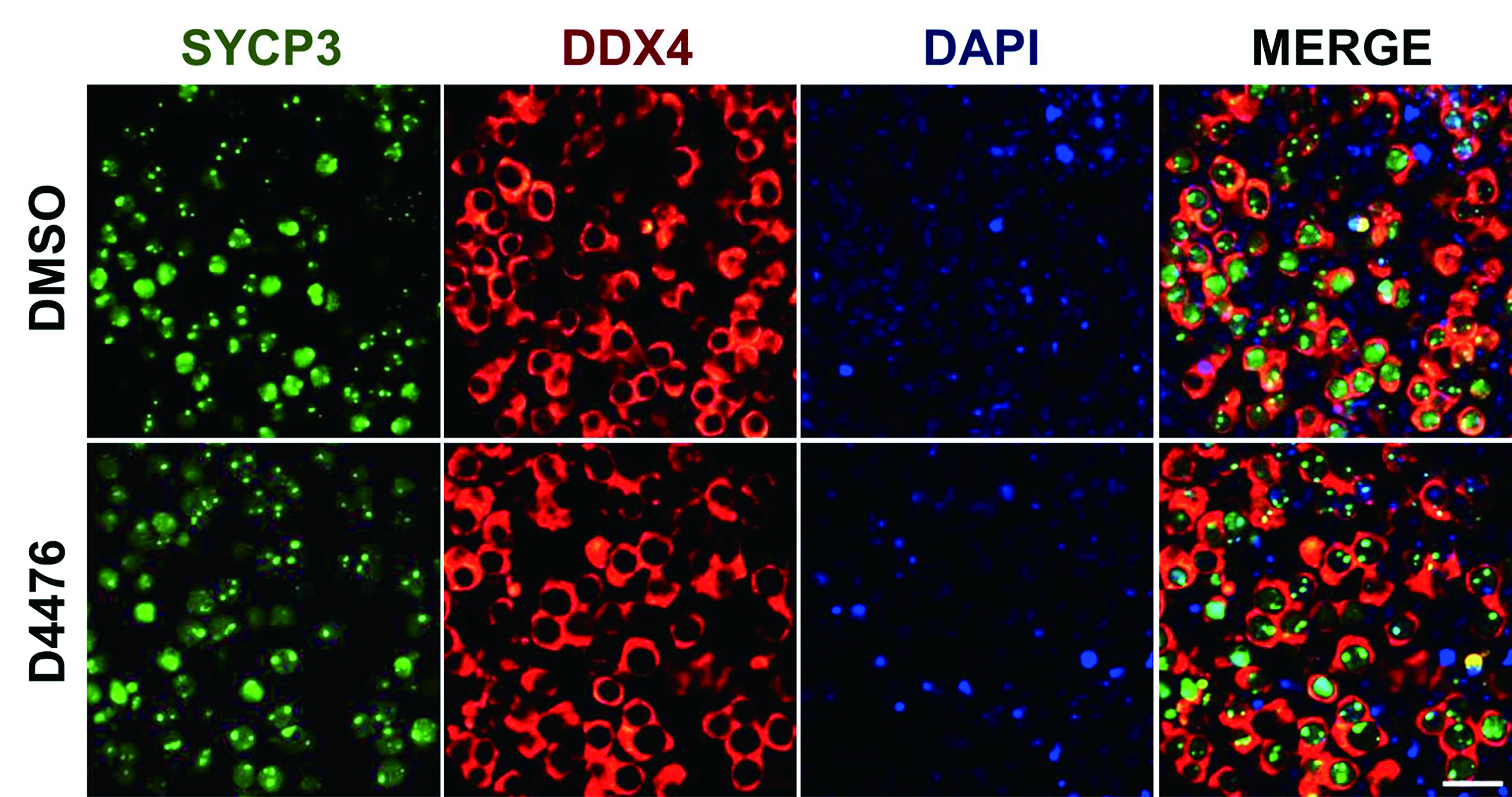

Supplement: Supplementary file 4 — Supplementary Figure 3 [file 41420_2022_1184_MOESM4_ESM.tif]

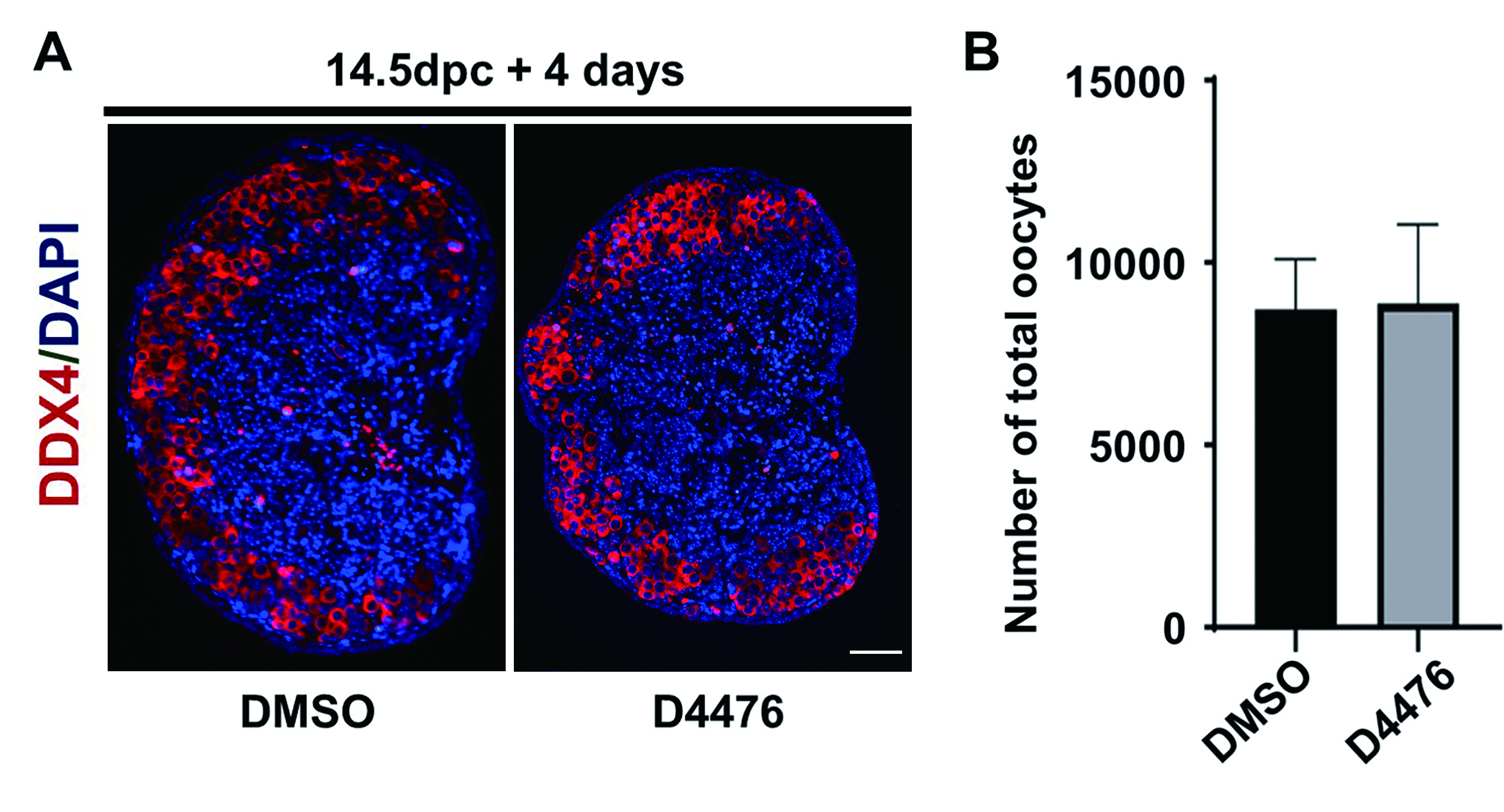

Supplement: Supplementary file 5 — Supplementary Figure 4 [file 41420_2022_1184_MOESM5_ESM.tif]

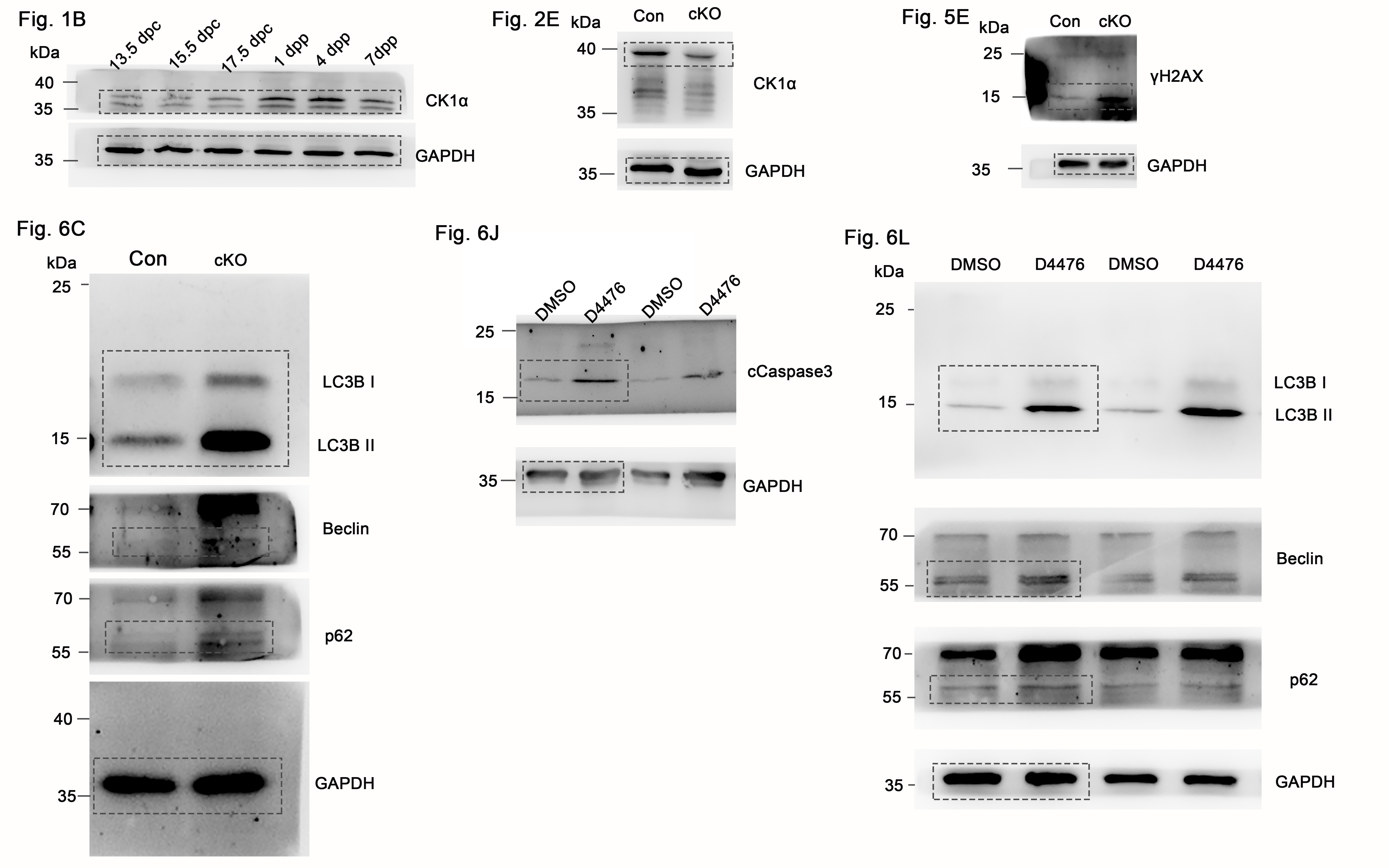

Supplement: Supplementary file 6 — Original Data File [file 41420_2022_1184_MOESM6_ESM.tif]
